# Seed germination and seedling growth of five desert plants and their relevance to vegetation restoration

**DOI:** 10.1002/ece3.4910

**Published:** 2019-01-22

**Authors:** Liming Lai, Lijun Chen, Mingqing Zheng, Lianhe Jiang, Jihua Zhou, Yuanrun Zheng, Hideyuki Shimizu

**Affiliations:** ^1^ Key Laboratory of Resource Plants, West China Subalpine Botanical Garden, Institute of Botany Chinese Academy of Sciences Beijing China; ^2^ Institute of Applied Ecology Chinese Academy of Sciences Shenyang China; ^3^ Information Center Ministry of Environmental Protection Beijing China; ^4^ National Institute for Environmental Studies Tsukuba Japan

**Keywords:** biomass allocation, germination, growth, horqin sandy land, precipitation, semiarid regions

## Abstract

Due to significant decreases in precipitation in northern China, knowledge of the response of seed germination and plant growth characteristics to key limiting factors is essential for vegetation restoration. We examined seed germination under different temperatures and water potentials, and we examined seedling growth under different amounts of water supply. Experiments were carried out in automatic temperature‐, humidity‐, and light‐controlled growth chambers. Under low water potentials, the final germination percentages of four herbaceous species were high, while seed germination of the shrub species *Caragana microphylla* was significantly inhibited. Under the different water supply amounts, seedlings of *Agropyron cristatum* allocated more biomass to the root and had a higher growth rate than those of *Elymus dahuricus* and *C. microphylla*. In light of these results and drier environmental conditions (annual mean precipitation is 366 mm, which falling mainly between June and August), potential selections for revegetation of different landscapes include the following: *A. cristatum* for shifting sand dunes, the establishment of the pioneer species *Agriophyllum squarrosum*,* C. microphylla* for semifixed sand dunes, *E. dahuricus* for fixed sand dunes, and *Melilotus suaveolens* and *Medicago sativa* for cultivation.

## 1 INTRODUCTION

In arid and semiarid lands, precipitation is usually low, and its distribution is unpredictable (Gutterman, 1993, 2002). Therefore, water is the most important restrictive environmental factor that determines the existence of plants. During the process of restoring ecosystems, vegetation rehabilitation has been found to be effective (Zheng et al., 2013). Furthermore, understanding plant species resistant to dry conditions is important for plant selections during the restoration.

The seed germination and early seedling growth are considered two critical phases for the successful establishment of a plant (Gutterman, 1993). Many plants have dormancy mechanisms that prevent germination until conditions are favorable for seed germination and seedling survival (Adondakis & Venable, 2004). Thus, germination characteristics can reflect the environmental conditions under which a species can be successfully established (Brändle, Stadler, Klotz, & Brand, 2003). In the field, temperature and moisture can limit germination of species and tolerance to these variables may be species dependent (Baskin & Baskin, 2014; Chauhan & Johnson, 2008). Therefore, knowing the thresholds and tolerances of different species is useful for informing revegetation plans. After seed germination, the seedling must develop quickly to overcome high mortality rates associated with this stage. Seedling survival of different species is threatened by a wide variety of factors, and drought is the primary limiting, especially in arid and semiarid areas where plant activity is tightly coupled with water availability (Moles & Westoby, 2004). Plants respond to specific water‐scarce environments with biomass allocation strategies to improve plant growth and the chances of survival (Padilla & Pugnaire, 2007). In arid regions where soil moisture is low near the soil surface, plants usually allocate more biomass to roots to increase water uptake (Padilla & Pugnaire, 2007; Pugnaire, Chapin, & Hardig, 2006).

The temperate steppe in China is one of the typical vegetation types in Asia and is thought to be highly sensitive to climate change, including significant changes in precipitation (Christensen, Coughenour, Ellis, & Chen, 2004). Located in this arid and semiarid land, the Horqin Sandy Land has undergone severe desertification over the past decades due to a series of human activities, such as overgrazing and intensive farming. During the vegetation restoration, establishing new populations with native plant species is an effective strategy (Abella, Craig, Smith, & Newton, 2012). The native species *Agropyron cristatum*,* Elymus dahuricus*,* Melilotus suaveolens*,* Medicago sativa*, and *Caragana microphylla* are widely distributed in the Horqin Sandy Land (Li, Ma, Xiao, Wang, & Kim, 2004). These species have high economic value in that they can be used as pasture plants, which can allow both vegetation restoration and grazing to occur on a grassland; thus, adopting these species as restoration plants can provide both benefits. The landscape of the Horqin Sandy Land, which includes moving, semifixed, and stabilized sand dunes, makes restoration complicated. Therefore, it is necessary to evaluate the germination and growth characteristics of these species to help with selecting the most appropriate for restoration on different sandy land, especially under drought and high fluctuations in temperature that occur in this environment.

The aims of this study were to investigate the effects of various temperatures and water potentials on seed germination and to evaluate growth characteristics under different water supply regimes. The following questions were posed: (a) How do the main limiting environmental factors affect seed germination and seedling growth? (b) What are the drought adaptabilities of the five species? We discuss these questions in the context of informing vegetation restoration.

## 2 MATERIALS AND METHODS

### 2.1 Study site

The Horqin Sandy Land is mainly located in the Northeast China, which belongs to semi‐arid zone (42°55′N, 120°41′E). The annual mean precipitation is 366 mm, with mainly occurring between May and September. The annual mean evaporation and temperature are approximately 1,935 mm and 6.8°C, respectively (Naiman Desertification Research Station, Chinese Academy of Sciences). The soils are susceptible to wind erosion and classified as *Cambic Arenosols* (Zhao et al., 2007).

### 2.2 Seed collections

Ripe seeds of five species (*A. cristatum*,* E. dahuricus*,* M. suaveolens*,* M. sativa,* and *C. microphylla*) were collected at the Horqin Sandy Land in 2004 (Table 1). For each species, a set of 20 square plots (100 m^2^ per plot) were set up. Seeds were collected from plants in each plot to obtain an adequate representation of genetic diversity. Seeds were stored in cloth bags at 4°C after cleaning and air‐dried.

**Table 1:** Characteristics of five species included in this study

Species name	Family	Ability to fix nitrogen	Life form	Regeneration
*Agropyron cristatum*	Gramineae	No	Perennial, grass	Seeds
*Elymus dahuricus*	Gramineae	No	Perennial, grass	Seeds
*Melilotus suaveolens*	Leguminosae	Yes	Annual, grass	Seeds
*Medicago sativa*	Leguminosae	Yes	Perennial, grass	Seeds
*Caragana microphylla*	Leguminosae	Yes	Perennial, shrub	Seeds

### 2.3 Experimental design

#### 2.3.1 Effects of temperature and water potential on germination

The experiments were conducted in automatic temperature‐, humidity‐, and light‐controlled growth chambers (KG‐306SHL‐D, Koito Co., Ltd., Tokyo, Japan). According to our previous study, seeds of these five species germinated best in the dark (Lai et al., 2016). Therefore, seed germination was tested in darkness.

For each species, treatment results from the combination of later potential level and temperature. In the field, seeds located at different sand layers were exposed to different moisture conditions. According to seed germination studies on plant of arid regions, seed can hardly germinate when water potential exceed −2.0 MPa (Baskin & Baskin, 2014); therefore, a series of water potentials including 0, −0.2, −0.4, −0.6, −0.8, −1.0, −1.2, −1.4, −1.6, −1.8, and −2.0 MPa were used to investigate germinate ability of the studied species. The water potential series were acquired with different concentrations of polyethylene glycol 6000 (PEG‐6000) solutions (Michel & Kaufmann, 1973). Experiments were conducted in darkness under 10, 15, 20, 25, and 30°C. These five temperatures are close to the spring and summer germination conditions in the study area, based on 30 years of observations (Qi, 1998). Seeds were sterilized with ultraviolet radiation. After that, seeds were placed on filter pater (three layers) on 90 mm diameter × 15 mm depth covered Petri dishes. Distilled water or solution of PEG‐6000 was added to the dishes until about half of the seeds were immersed. There were five replicate of 25 seeds for each treatment of studied species. Seeds were investigated daily under 10 µmol m^−2 ^s^−1^ light. Seed was considered germinated with emergence of radicle (Baskin & Baskin, 2014), and it was discarded after counting. Experiments were continued for 30 days (Zheng, Xie, Gao, Jiang et al., 2005).

In this experiment, two indices were used to measure seed germination: the final germination percentage (GP) and germination rate (GR). The GR is calculated with the modified Rozema index of germination rate (Rozema, 1975): $\sum \frac{100G_{i}}{nt_{i}}$, where *n* is the number of seeds in a treatment and *G*_i_ is the number of seeds germinated on the day *t*_i_ (*t*_i _= 0, 1, 2, 3…).

#### 2.3.2 Effect of the water supply regime on plant growth

The experiments were conducted in automatic temperature‐ and humidity‐controlled growth chambers under natural light conditions (S‐203A, Koito Co., Ltd. Japan). The humidity of the chambers was set to 60%:50% (night:day). The temperature was set to 15:25°C (night:day), which is the optimal temperature for growth of the tested species according to our previous study (Lai et al., 2016).

Seeds were sown in pots made of PVC (11.2 cm in diameter and 20 cm in height). The experiment began 4 weeks after seed germination. The pots were filled with prepared sand, which was mixed from different particle size groups according to the proportions of those under field conditions in the study area. There were 10 replicates (each consisting of one pot with one seedling) per treatment for each species. The pots were watered with distilled water immediately after the seeds were sown. After 4 weeks' seedling nursery since seed germination, the water supply treatments were applied. At starting of the experiment, 10 seedlings of each species were harvested to get the primary values that used for growth analysis. For each species, four water supply treatments were applied: 3, 6, 9, and 12 mm per 3 days, which is equivalent to 30, 60, 90, and 120 mm each month, respectively. The experiment lasted for 30 days following treatment application.

In this experiment, the net assimilation rate (NAR) and the relative growth rate (RGR) of were calculated with the equations:

(1) $$NAR=\frac{1}{t_{2}-t_{1}}\int_{w_{1}}^{w_{2}}\frac{1}{s}\frac{\mathrm{dw}}{\mathrm{dt}}dt=\frac{\left(w_{2}-w_{1}\right)\left(\ln s_{2}-\ln s_{1}\right)}{\left(s_{2}-s_{1}\right)\left(t_{2}-t_{1}\right)}$$

(2) $$RGR=\frac{1}{t_{2}-t_{1}}\int_{w_{1}}^{w_{2}}d\left(\ln w\right)=\frac{\ln w_{2}-\ln w_{1}}{t_{2}-t_{1}}$$

where *w*_1_ and *s*_1_ are the dry mass of whole plant and total leaf area at the beginning time of the investigation (*t*_1_), respectively, and *w*_2_ and *s*_2_ are the dry mass of whole plant and total leaf area at the final harvest (*t*_2_), respectively (Xiong, Mueller, & Day, 2000).

Following variables were calculated from the primary data: the increased dry weight percentage (IDW, increase in total dry weight since treatment application/total dry weight at the end; in g/g), dry weight of aboveground biomass/dry weight of belowground biomass (S/R ratio, in g/g), leaf area ratio (LAR; leaf area/total plant mass, in m^2^/kg), and specific leaf area (SLA; leaf area/leaf mass, in m^2^/kg).

### 2.4 Statistical analysis

For each species, effects of temperature and water potential on final GP were analyzed with generalized linear models (GLM) with a Poisson distribution, where the effects on germination rate were analyzed with GLM with a Gaussian link function. Data of growth variables including IDW, S/R, LAR, SLA, NAR, and RGR were an analyzed with GLM with a Gaussian link function. Data were log‐transformed if necessary (Lindgren, Ove Eriksson, & Jon Moen, 2007). All statistical analyses were performed using SPSS 13.0 (SPSS, 2004).

## 3 RESULTS

### 3.1 Effects of temperature and water potential on seed germination

The main effects of temperature and water potential had significant effects on the GP and GR of all five species (Table 2).

**Table 2:** The effects of temperature (*T*) and water potential (WP) on seed germination, analyzed with a generalized linear model with Poisson distribution for final germination percentage (GP) and Gaussian distribution for germination rate (GR) for the five species

Source	*df*	*Agropyron cristatum*		*Elymus dahuricus*		*Melilotus suaveolens*		*Medicago sativa*		*Caragana microphylla*	
		FG	GR	FG	GR	FG	GR	FG	GR	FG	GR
*T*	4	368.8**	738.0**	46.4**	2430.1**	159.3**	387.9**	47.8**	955.9**	81.3**	388.2**
WP	10	58.3**	113.1**	50.5**	185.5**	233.6**	217.3**	31.3**	141.5**	245.3**	355.3**

Notes

Data represent Wald chi‐Square values.

** *p* < 0.01.

For these two *Gramineae* species, temperature showed stronger effects on seed germination than those of water potential.

**Figure 1:**
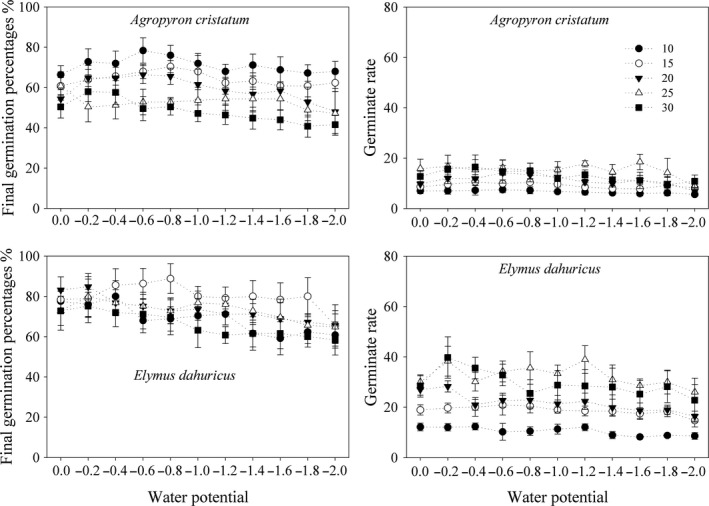
Final germination percentages and germinate rates (mean ± 95% CI) of two *Gramineae* species under different temperatures and water potentials

For *A. cristatum*, seed germination was less responsive to water potential, lower temperature can promote GP but decrease GR (Figure 1). In detail, the GP was higher under the lower temperature of 10°C than that under the higher temperatures, especially 30°C. The decreasing of water potential from 0 to −0.6 Mpa increased GP. The responses of the GR to temperatures contradicted those of the GP. At the same water potential, seeds germinated faster under higher temperatures (25 and 30°C).

For *E. dahuricus*, responses of seed germination to temperature and water potential were similar as those of *A. cristatum* in most conditions (Figure 1). In detail, the GP was higher under the lower temperature of 15°C than under the other temperatures. The highest and lowest GPs were 78.4 ± 3.7% and 41.6 ± 1.6% for *A. cristatum* and 88.8 ± 2.7% and 58.4 ± 2.7% for *E. dahuricus*, respectively.

For these three Leguminosae species, both temperature and water potential showed important effects on germination of *C. microphylla*, and water potential had stronger effects on germination of *M. suaveolens*, while temperature had stronger effects on GRs but not the GPs of the *M. sativa*.

**Figure 2:**
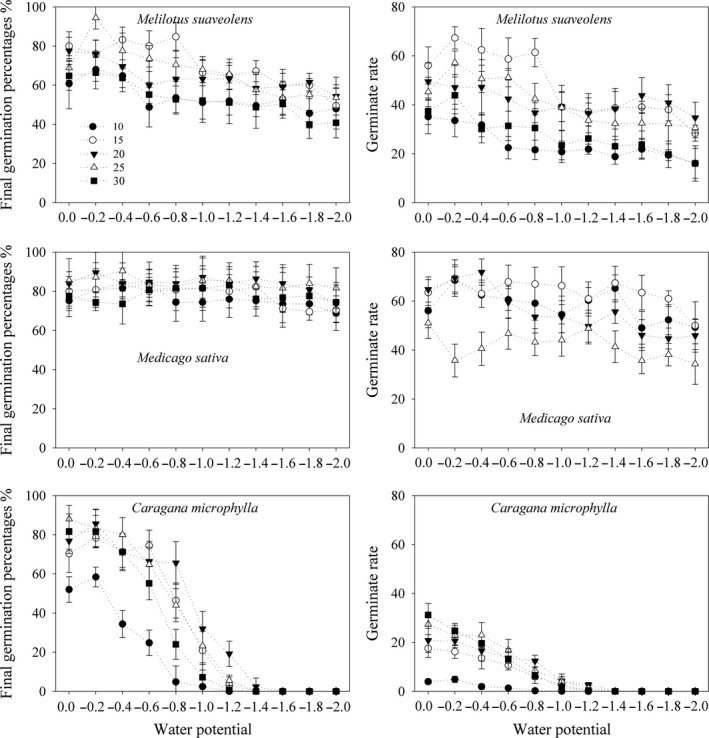
Final germination percentages and germinate rates (mean ± 95% CI) of three Leguminosae species under different temperatures and water potentials

In detail, seeds germinated to higher GPs under the medium temperatures (15, 20, and 25°C; Figure 2). The decrease in water potential from 0 to −0.2 MPa increased the GPs of these three species under almost all temperature treatments. A further decrease in the water potential significantly reduced the seed germination of *M. suaveolens* and *C. microphylla*, but similar effects were not found for the seed germination of *M. sativa*. When the water potential reached −1.0 MPa and lower, the GPs of *M. suaveolens* and *M. sativa* were significantly higher than that of *C. microphylla* under the same temperature. The highest and lowest temperature in the experiment decreased the seed germination rate, especially the lowest temperature. The GRs of *M. suaveolens* and *M. sativa* were significantly higher than that of *C. microphylla* under the same temperature. Under the same temperature, the GR was reduced with further decreases in water potential from −0.2 MPa.

### 3.2 Effect of the water supply regime on plant growth

In general, the water supply regime had significant effects on all growth variables of the five species (Table 3). Besides the biomass index IDW, the RGR was closely correlated with SLA for all five species (Table 4). For *A. cristatum*, seedling growth was greatly stimulated with more water addition, which had the highest IDW, NAR, and RGR among five species. For *E. dahuricus*, the stimulating effects were lowest among five species that had lowest IDW, NAR, and RGR. For *M. suaveolens*, water addition stimulated IDW increasing to higher values that only less than those of *A. cristatum*, but the S/R ratio was unresponsive to water treatment. For *M. sativa*, SLA values were higher and decreased with more water addition. For cm, the S/R ratio was unresponsive to water treatment.

**Table 3:** Results of generalized linear model with Gaussian distribution on growth parameters of the five species under different water supply regimes

Variables	*df*	*Agropyron cristatum*	*Elymus dahuricus*	*Melilotus suaveolens*	*Medicago sativa*	*Caragana microphylla*
IDW	3	87.5**	624.5**	391.4**	79.3**	52.2**
S/R	3	10.2*	17.7**	17.7**	15*	6.1^ns^
LAR	3	5.9 ^ns^	22.1**	22.1**	22.4**	30.2**
SLA	3	1.8 ^ns^	229.4**	229.3**	245.2**	12.6**
NAR	3	18.8**	129.1**	129.1**	110.9**	70.3**
RGR	3	86.5**	231.4**	231.4**	191.0**	50.9**

Data represent Wald chi‐square values.

IDW: increased dry weight percentage; LAR: leaf area ratio; NAR: net assimilation rate; ns: not significant; RGR: relative growth rate; S/R: ratio of shoot dry weight and root dry weight; SLA: specific leaf area.

* *p* < 0.05.

** *p* < 0.01.

**Table 4:** Pearson correlation coefficients between RGR and other growth indexes for five species

	IDW	S/R	LAR	SLA	NAR
*Agropyron cristatum*	0.99**	0.74	0.38	−0.92*	0.81
*Elymus dahuricus*	0.99**	0.71	0.97*	0.99**	0.99**
*Melilotus suaveolens*	0.99**	−0.13	−0.96*	−0.99**	0.99**
*Medicago sativa*	0.99**	0.92*	0.94*	−0.93*	0.98*
*Caragana microphylla*	0.99**	0.94*	−0.98*	−0.94*	0.99**

Abbreviations are same as Table 3.

* *p* < 0.05.

** *p* < 0.01.

**Figure 3:**
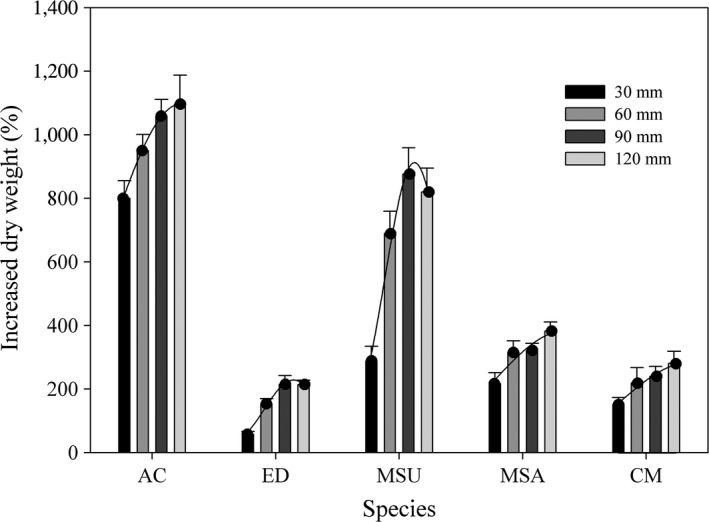
Increased dry weight percentage (mean ± 95% CI) of five species under different water supply regimes. AC: *Agropyron cristatum*; CM:* Caragana microphylla*; ED: *Elymus dahuricus*; MSA:* Medicago sativa*; MSU:* Melilotus suaveolens*. The Gaussian or Quadratic curve was used to fit the relationship between water supply regimes and increased dry weight percentage (*p < *0.05)

In detail, a greater water supply can significantly stimulate seedling growth, and the IDW was the highest under a 90 or 120 mm water supply regime for all species. Under the different water supply treatments, the IDW values of *A. cristatum* were significantly higher than those of the other four species, followed by *M. suaveolens*,* M. sativa*,* C. microphylla,* and *E. dahuricus* (Figure 3).

**Figure 4:**
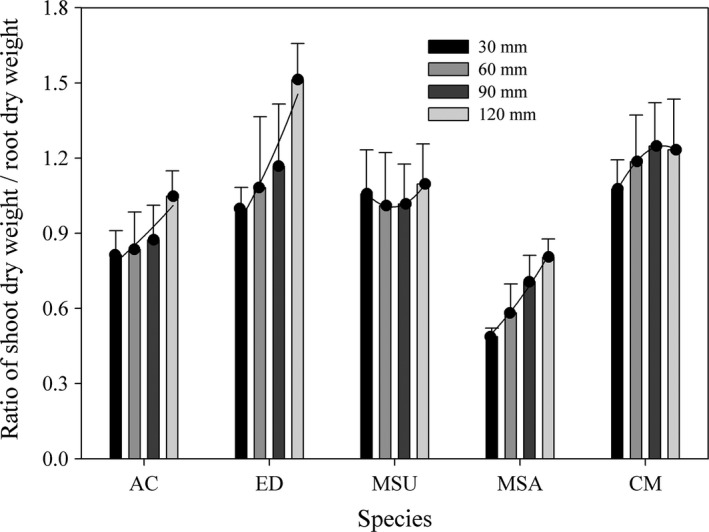
Ratio of shoot dry weight/root dry weight (mean ± 95% CI) of five species under different water supply regimes. Other descriptions are same as Figure 3

The S/R ratio increased with a greater water supply. However, there were no significant differences among the S/R ratio values under different water supply treatments for *M. suaveolens* and *C. microphylla* (Figure 4). For the two Gramineae species, the S/R ratio was lower for *A. cristatum* (0.80 ± 0.04 to 1.1 ± 0.04) than for *E. dahuricus* (0.99 ± 0.04 to 1.51 ± 0.06); for the three Leguminosae species, the S/R ratio was highest for *C. microphylla* and lowest for *M. sativa*.

**Figure 5:**
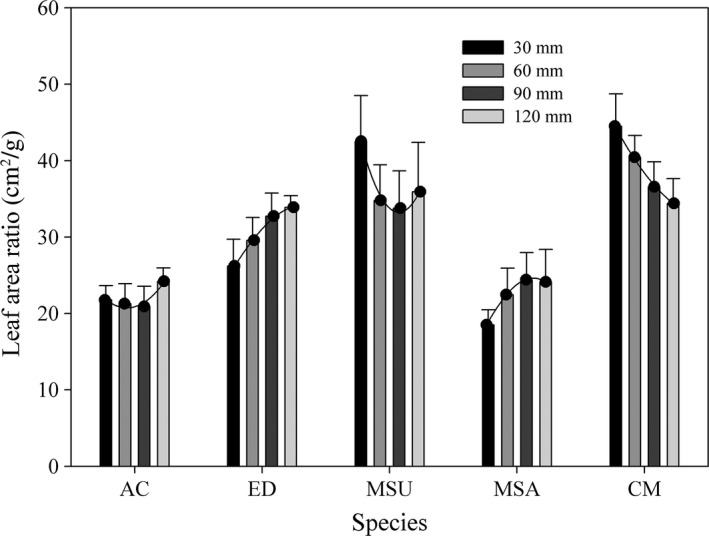
Leaf area ratio (mean ± 95% CI) of five species under different water supply regimes. Other descriptions are same as Figure 3

The water supply regime had less effect on the LAR for *A. cristatum*, and the values were lowest among five species under same treatment (except LAR for MSA under 30 mm). For *E. dahuricus* and *M. sativa*, the LAR increased with increasing water supply, but the opposite trend was found for *C. microphylla* (Figure 5).

**Figure 6:**
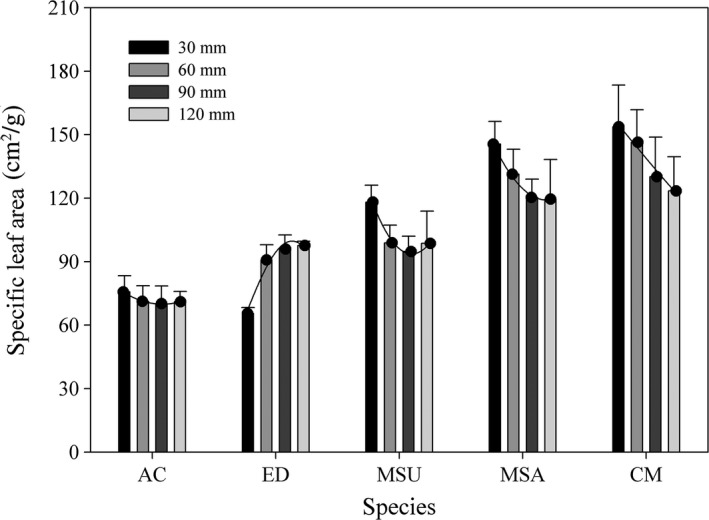
Specific leaf area (mean ± 95% CI) of five species under different water supply regimes. Other descriptions are same as Figure 3

The SLA values for the three Leguminosae species were higher than those for the two Gramineae species under the same water treatment. The different water supply regimes did not affect the SLA for *A. cristatum*, the 30 mm water supply significantly decreased the SLA for *E. dahuricus*. But the three Leguminosae species showed an opposite responses that more water addition decreased SLA (Figure 6).

**Figure 7:**
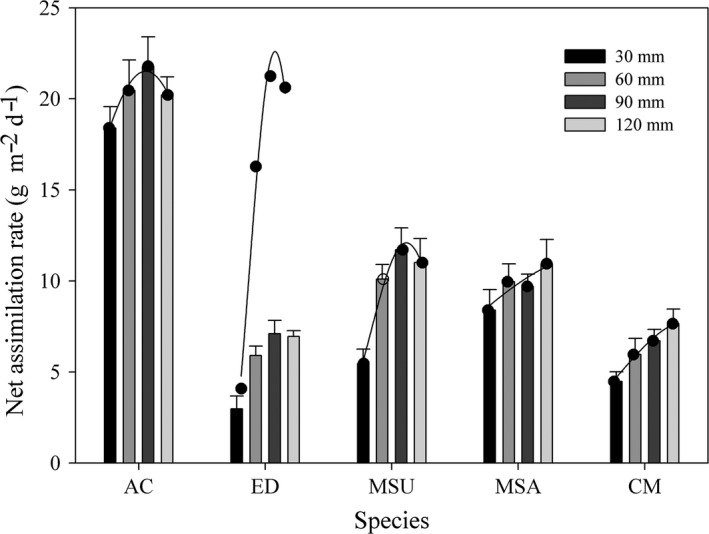
Net assimilation rate (mean ± 95% CI) of five species under different water supply regimes. Other descriptions are same as Figure 3

In general, the NAR for all five species increased with a greater water supply regime, and the NAR values under the 30 mm water supply were significantly lower than those under the other water supply treatments. When the water supply regime increased from 90 to 120 mm, the NAR did not change significantly. The NAR was highest for *A. cristatum*, intermediate for *M. suaveolens* and *M. sativa*, and lowest for *E. dahuricus* and *C. microphylla* (Figure 7).

**Figure 8:**
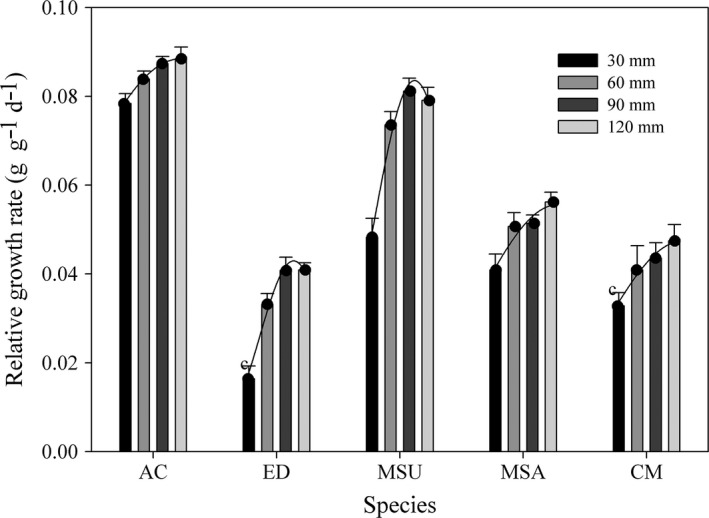
Relative growth rate (mean ± 95% CI) of five species under different water supply regimes. Other descriptions are same as Figure 3

The RGR for all five species increased with a greater water supply regime, and the values under the 30 mm water supply were significantly lower than those under the other water supply treatments (Figure 8). Under the different water supply treatments, *A. cristatum* had a significantly higher RGR than did the other species under the same water supply regime, and the highest value was 0.089 ± 0.001 g g^−1^ day^−1^, which was observed in the 120 mm water supply. *Elymus dahuricus* had the lowest RGR values among the four species, which range from 0.016 ± 0.001 to 0.041 ± 0.001 g g^−1^ day^−1^.

## 4 DISCUSSION

During the whole life of a plant, seed is the stage that has greatest tolerance to environmental stresses; however, seedling stage is the most sensitive period (Gutterman, 1993). In the study, different responses to temperature and water treatments were investigated during seed germination and seedling growth stages, which can be important for appropriate species selections.

### 4.1 Response of seed germination to temperature and water potential

Seeds of plant species distributed in typical of arid regions can germinate within the temperature range of 5–40°C (Gutterman, 1993). In this study, the GPs of the five species showed different patterns under the five constant temperatures. First, seed germination of *E. dahuricus* and *M. sativa* reached higher GPs under all five test temperatures. Second, the GP of *A. cristatum* decreased with increasing temperature; in contrast, the GP of *C. microphylla* increased with increasing temperature. Third, seed germination of *M. suaveolens* was stimulated under medium temperatures and inhibited under the highest or lowest temperatures. In the Horqin Sandy Land, the mean temperatures of the soil surface are 1.1, 12.3 and 20.8°C in March, April, and May, respectively (Zhang, Zhao, Zhang, Zhao, & Drake, 2005). According to the GP of these species, *A. cristatum* can germinated to higher percentages under lower temperature; *M. suaveolens* and *C. microphylla* prefer 15 and 25°C, respectively; while *M. sativa* and *E. dahuricus* could germinated at all ranges of tested temperatures. For seed sown time of these five spring‐germinating species, middle‐April is proper for of *A. cristatum*, late‐April and early‐May are proper for *M. suaveolens* and *C. microphylla*, and seed germination of *M. sativa* and *E. dahuricus* have less temperature restrictions in this season.

Many studies have been performed on seed germination responses of arid‐region species to water potential, and some species cannot germinate effectively even at –0.2 MPa (Zheng, Xie, Gao, Yu et al., 2005). However, some species of arid regions can germinate to high final percentages under water potentials lower than −0.8 MPa (Tobe, Li, & Omasa, 2000; Zheng et al., 2004). In this study, germination of the five species differed in response to water potential. The shrub species *C. microphylla* was significantly inhibited by lower water potential. However, the four herbaceous species showed higher adaptabilities to decreased water potential, especially *M. sativa*: the GPs under all five temperatures exceeded 70%. These results indicated that the four herbaceous species could germinate under very dry conditions.

In the field, a frequent hazard that a germinating seed is likely to face is periods of drought before the process is complete (Fenner & Thompson, 2005). Especially in arid and semiarid region, the long and intermittent drought periods are fatal to seed germination. In addition to water requirements for germination, some species also have mechanisms that ensure seeds produced by a single individual germinate at more than one time (Adondakis & Venable, 2004). In our study, the GR for all five species showed a descending trend with a decrease in water potential. This finding indicated that a lower GR was a mechanism allowing seeds to protect themselves in environments with drought. Among the five species, *M. suaveolens* and *A. cristatum* had the fastest and the slowest GR, respectively (in conditions under which *C. microphylla* can germinate normally). These GR patterns may determine which species will establish. The slow GR strategy allows the germination of *A. cristatum* to occur cumulatively and is advantageous for its survival in the environments where rain events are of short duration. This may be one reason that *A. cristatum* is distributed on the semifixed sand dunes (Li et al., 2004). However, the fast germination needs a steady soil moisture environment that can promote the newly emerged seedlings grow to a proper size to withstand the subsequent dry period (Fenner & Thompson, 2005). Thus, though the other three herbaceous species can germinate to high GPs, the faster GR may restrict survival and allow fast germinators to be distributed in the drier environment.

### 4.2 Effect of water supply on seedling growth

Mortality is usually high during the period between seed germination and the establishment of a juvenile plant, and this may act as a strong selective filter of seedling growth traits (Moles & Westoby, 2004).

Generally, drought can reduce the biomass of plants in the field and greenhouse (Heilmeier et al., 2002; Zheng et al., 2013). However, differences in the response of dry weight to increased water supply existed among these species. The dry weight of the studied species increased significantly with an increase in the water supply from a low amount (30 mm) to medium amounts (60 and 90 mm), but more water did not increase the dry weight, except *M. sativa* that dry weight was significantly increased when water amount reached 120 mm. This finding indicated that when there is sufficient water, other environmental factors, such as soil nutrients, may become the main limiting factor of plant growth; while for *M. sativa*, biomass production was limited only by water in this study.

To improve plant growth and the chance of survival in specific water‐scarce environments, different biomass allocation strategies may be used by plants (Padilla & Pugnaire, 2007). In arid regions where soil moisture is low near the soil surface, more biomass is allocated to roots to increase water uptake (Padilla & Pugnaire, 2007; Pugnaire et al., 2006), but in places with sufficient water, plants tend to allocate more to aboveground parts (Heilmeier et al., 2002). Thus, plants in drought‐prone regions are expected to have a low ratio of shoot dry weight/root dry weight (Zheng et al., 2013). In this study, the S/R ratio showed a significant increase when the water supply reached 90 mm for *A. cristatum*,* E. dahuricus,* and *M. sativa*. This finding may suggest that for these species, 90 mm/month precipitation can relieve the effect of drought stress on plant survival, and more biomass can be allocated to photosynthetic organs to accelerate growth and quickly capture the nutrient resources that are limited under suboptimal conditions (Heilmeier et al., 2002).

At suboptimal conditions, plant species show wide variation in RGR (Grime & Hunt, 1975; Poorter, 1999). It was stated previously that there is a positive correlation between the RGR of a plant species and its occurrence in nutrient‐rich habitats and that this relationship is caused by selection for high RGR in a productive environment and low RGR under unproductive conditions (Grime, Cornelissen, Thompson, & Hodgson, 1996). In our experiment, similar results were found, namely, the RGR of all five species was lowest in the 30 mm/month water supply treatment, and the values significantly increased with increasing water supply under most conditions (Figure 8). Additionally, the RGR achieved under favorable conditions can be considered an indication of the potential ability of a species to take advantage of favorable growth opportunities, and this can be thought of as the growth strategy of a species (Wright & Westoby, 2001). The stimulating effects on RGR by an increase in water supply were larger for *E. dahuricus* and *M. suaveolens*, and this indicates that in the field, these two species are better able to grow in a water‐sufficient environment. Among five species, *A. cristatum* had the highest RGR and was special for its high and close RGR values under different water supply treatments, though there were significant differences among the RGR values. These results indicate that seedlings of *A. cristatum* have higher tolerance under different soil water conditions, and it could make use of the limited precipitation to accelerate its growth to finish establishment (Padilla & Pugnaire, 2007). Also, these may explain why *A. cristatum* can be distributed in semifixed sand dunes and becoming a widespread invasive species in many arid regions (Sheley, Mangold, & Anderson, 2006; Steers & Allen, 2010).

Because the RGR is an essential attribute for performance of plant species in natural habitats, many studies have been conducted on the correlations between the RGR and other growth indexes (Shipley, 2002; Wright & Westoby, 2001). Grubb, Lee, Kollman, and Wilson (1996) stated that the RGR is first determined mainly by SLA, and then, the more important determinant of RGR changes in older seedlings. In this study, the result was consistent with results reported by others (Grubb et al., 1996; Shipley, 2002).

### 4.3 Selection of the five species for vegetation restoration

In addition to their high economic value as drought tolerant forage, there are many reports of the applications of these five species for restoration. *Agropyron cristatum* is considered an invasive species in the ecological restoration process (Ambrose & Wilson, 2003); *E. dahuricus*,* M. sativa* and *M. suaveolens* are widely used in the restoration of degraded fields on the Loess Plateau, China (Li, Xu, & Wang, 2008; Li & Zhang, 1991); and *M. sativa* and *C. microphylla* are used as air‐seeding species in the Ordos Plateau (Zheng et al., 2003).

The landscape of the Horqin Sandy Land is complex because it includes shifting, semifixed, and fixed sand dunes. Based on the establishment of the environments, the succession of vegetation can be summarized as follows: first, pioneer species on bare shifting sand dunes; second, establishment of secondary species on semifixed sand dunes; and last, establishment of climax species on fixed dunes (Liu, 1985). As discussed above, for the seed germination stage, a higher final GP and low germinate rate are important criteria for selecting species to be used for restoration of sandy environments; after seed germination, seedlings with a lower S/R ratio, and higher RGR and NAR can utilize belowground water more efficiently and grow faster, which provides better resistance to drought (Maun, 1998). Among the five species, *A. cristatum* has the best ability to establish on sandy land; however, the lower root distribution depth and tendency to be buried by sand may limit the distribution of this species on bare, shifting sand dunes (Ambrose & Wilson, 2003; Maun, 1998). Thus, *A. cristatum* can be used for restoration of shifting sand dunes after the establishment of the pioneer species *Agriophyllum squarrosum*, as was also found in a field survey (Liu, 1985). The vegetation coverage and soil moisture content of semifixed and fixed sand dunes are better than those of shifting sand dunes. The shrub species *C. microphylla* can better resist wind‐blown sand and sand burial because individuals of this species are taller with stronger stems, and their greater root distribution depth can help them access deeper soil water (Li et al., 2008). Thus, *C. microphylla* is suitable for use on semifixed sand dunes. *Elymus dahuricus* had a medium seed germination rate but lower RGR and NAR, and it can be used on fixed dunes. Seeds of *M. suaveolens* and *M. sativa* germinated rapidly, a characteristic that requires better moisture conditions for supporting seedling survival. Moreover, we found in this study that both the RGR and NAR of these two species were significantly reduced under drier conditions (30 mm water supply); this finding also indicated that seedling growth of these species requires a greater water supply such as that provided by manual irrigation. In fact, these two species are commonly cultivated for pasture usage; thus, both of them would be suitable for cultivation on fixed sand dunes.

Due to different germination and seedling growth types, the seed dispersal time should be carefully considered. Based on climatic data analysis of the Horqin Sandy Land (Zhang et al., 2005), *A. cristatum* is suitable for sowing in early March to ensure a lower‐temperature environment. The monthly mean precipitation in the study area increased from 14.1 mm in April to 35.7 mm in May. To ensure the survival of seedlings, *E. dahuricus* and *C. microphylla* could be sown in early April to select a warmer and moister climate. The higher amount of precipitation in May is beneficial to seedling growth and survival of *M. suaveolens* and *M. sativa* since they are fast germinators.

## 5 CONCLUSION

In this study, we evaluated the seed germination and seedling growth responses of five key plant species of desertified areas to explore the applications of these species for vegetation restoration. Under different temperature treatments, the seed germination of *A. cristatum*,* M. suaveolens,* and *C. microphylla* was significantly affected, but *E. dahuricus* and *M. sativa* show a lower range of variation. The shrub species *C. microphylla* was significantly inhibited by lower water potential. However, the seed germination of four herbaceous species showed higher tolerance to decreasing water potential. The seedling growth of *A. cristatum* showed higher drought resistance than did that of *E. dahuricus* and *C*. *microphylla*. During restoration, seed germination and seedling growth characteristics should be considered for the specific landscape.

## CONFLICT OF INTEREST

None declared.

## AUTHOR CONTRIBUTIONS

Liming Lai, Yuanrun Zheng: Wrote the paper. Lijun Chen: Performed the experiments. Mingqing Zheng, Lianhe Jiang, Jihua Zhou: Analyze the data. Yuanrun Zheng, Hideyuki Shimizu: Conceived and designed the experiments.

## Data Availability

The data including the raw dataset will be archived in the Dryad after acceptance of the paper.
